# Dust Mites Population in Indoor Houses of Suspected Allergic Patients of South Assam, India

**DOI:** 10.5402/2011/576849

**Published:** 2011-06-07

**Authors:** Dhruba Sharma, B. K. Dutta, A. B. Singh

**Affiliations:** ^1^Department of Botany, Rajiv Gandhi University, Rono Hills, Arunachal Pradesh, Doimukh 791112, India; ^2^Department of Ecology and Environmental Science, Assam University, Dorgakona, Silchar 788011, Assam, India; ^3^Institute of Genomics and Integrative Biology, CSIR, Delhi University Campus, Mall Road, Delhi 110 007, India

## Abstract

*Background*. In the present study, quality and quantity of indoor dust mites was evaluated at the residence of 150 atopic allergic patients from four different districts of South Assam. *Methods*. Suspected patients with case history of allergic disease were selected for indoor survey. Dust samples (500 mg) were collected from the selected patient's house and were analyzed using standard methods. *Results*. About 60% of the selected patients were found suffering from respiratory disorders and rest 40% from skin allergy. The dominant mites recorded from indoor dust samples were *Dermatophagoides* followed by *Blomia*, *Acarus*, and *Cheyletus* while *Caloglyphus* was recorded in least number. The distribution of mites on the basis of housing pattern indicates that RCC type of buildings supports maximum dust mite's population followed by Assam type (semi-RCC) buildings, and the lowest count was observed in wooden houses. Environmental factors like temperature, rainfall, and relative humidity are found to determine the indoor mite's population. Severity of allergic attack in some of the typical cases was found to be proportional to the allergen load of mites in the dust samples. *Conclusions*. The economic status, housing pattern, and local environmental factors determine the diversity and abundance of dust mites in indoor environment.

## 1. Introduction

One of the most strongly allergenic components of house dust, often heavily contaminated with the fecal pellets and cast skins, is house dust mites [1]. House dust mites are tiny creatures related to ticks, chiggers, and spiders that live in close association with humans. Their primary food is dander (skin scales) shed from human and pet activity [2]. Estimates are that dust mites may be a factor in 50 to 80 percent of asthmatics, as well as in countless cases of eczema, hay fever, and other allergic ailments. Symptoms are usually respiratory in nature (sneezing, itching, watery eyes, wheezing, etc.); however, there are reports of a red rash around the neck. Other allergic reactions may include headaches, fatigue, and depression [3]. Inhalations of dust mite allergens by hypersensitive individuals can result in acute attacks of bronchial asthma, accompanied by wheezing, shortness of breath, and perhaps even death. Diagnostic tests and clinical studies by allergists have shown house dust mite to be the most common allergy in asthmatics, and an important “root cause” for the development of asthma in young children. Recent studies suggest that at least 45 percent of young people with asthma are allergic to house dust mites. Unlike “seasonal” allergies caused by molds and pollen, people who are allergic to dust mites often will have symptoms year round [4]. The role of house dust mites in inducing allergy has been increasingly recognized by allergologist and aerobiologists. However, clinical investigations of house-dust mite allergy in tropics are few [1, 5–7]. The significant role of mites in the house dust responsible for health hazard such as respiratory allergy, nasobronchial, nasal, and skin allergy in sensitive individuals is well documented. Group 1 allergens of the mites *Dermatophagoides farinae* (*Der* f1) and *Dermatophagoides pteronyssinus* (*Der* p1) are the most significant allergens; 80% to 95% of patients allergic to dust mites have an elevated IgE response to them [8].

Shivpuri et al. were the first Indian acarologists to conduct extensive and intensive studies on mites and recorded that mites could grow well in house dust at 25°C temperature and 80% relative humidity [9–11]. Besides West Bengal [12, 13], and Maharastra [14–16], no much work on indoor dust mites survey has been carried out in other parts of India. In the present study, an attempt has been made to investigate the variety and variability of dust mites in the floor and bed dust of atopic allergic patients and to correlate it with the severity of allergic diseases in selected patients from Assam which is situated in the Northeastern part of India. The average atmospheric temperature and relative humidity of this part of India ranges from 29 to 32°C and 90 to 94%, respectively.

## 2. Materials and Methods

In the present study, only soft cell mites present in the house dust samples were selected. Population of dust mites at the residence of 150 atopic patients from Assam was surveyed from January to December 2005, to record the diversity and abundance of house dust mites in floor and bed dust samples. Allergic diagnostic profile was developed through standard questionnaires developed in the format provided by Patel Chest Institute, Delhi, to categorize the patients on the basis of age, sex, symptoms, housing patterns, and so forth. Patients of different economic status, age, and sex were selected for investigation. Of the total population selected, 47% were living in RCC type buildings (buildings with concrete wall, floor, and roof), 39% in Assam type (semi-RCC buildings having roof made of tin metallic element) buildings, 13% in bamboo house, and rest 7% in wooden houses. Sex wise, 73% of the selected patients were male and 27% were female. Only those patients who were having case history of allergic diseases (i.e., respiratory and skin allergic diseases) were selected for present study. Patients were identified and categorized after verifying their medical reports. Allergy confirmation reports in certain cases were found doubtful due to which all the selected patients were grouped as suspected allergic patients.

Five hundred milligrams (500 mg) of the indoor house dust sample were collected from both the floor and bed of the selected patient's house. Since the vacuum cleaners are not used in majority of Indian homes, therefore, dust sample was collected manually following the method described by Tilak et al. [2]. Patients were advised not to clean their floor and sleeping bed for two days before collecting the dust sample. Samples were collected in autoclaved plastic container. Large particles and fibrous materials in the dust were separated by sieving through 300 mess brass sieve of 6 mm diameter. Mites were isolated from the dust sample manually with the help of a painting brass (no. 6). Isolated mites were made clear by putting in 50% lactic acid for 24 hrs. Then they were mounted in the centre of a glass slide with a drop of melted glycerine jelly [17]. The presence of house dust mites was confirmed by examining them under the microscope. Mites types were categorized on the basis of genus, species, and their gender. The identification of house dust mites was done with the help of reference slides and literatures available [17, 18]. About 48.6% of the patients reported maximum allergic symptoms during winter, while 21.4% suffer maximum during summer. However, 25.7% patients reported allergic problem round the year irrespective of season. The meteorological data was collected from the Tocklai research center, Silchar.

An attempt has been made to correlate the total mites population recorded in the dust samples of each patient with the severity of their allergic attack. On the basis of the allergic attack, patients were graded under four grades as follows.

G1: Occasional skin allergy attack
G2: Frequent skin allergy attack
G3: Occasional respiratory attack
G4: Frequent respiratory attack.

Samples were categorized on the basis of allergic symptoms, housing pattern, seasons, and surface types (bed/floor). Correlation analysis was done to examine the relationship between allergic/respiratory symptoms and indoor dust mite's population. To explore the relationship between mite counts and RH and temperature, a simple product-moment correlation analysis was conducted. 

Statistical analysis like mean, correlation, and regression were carried out using MINITAB 11.2 and ORIGIN 7.0 statistical software.

## 3. Results

Fifty percent of the total patients selected for indoor mites survey were of the age group between >30–50 yrs, and 31% were of the age group upto 30 yrs, while the rest 20% were of the age group of >50 yrs. Sex wise, 73% of the patients were male and the rest 27% were female. About 60% of the patients were reported to be suffering from respiratory allergic disorders, and the rest 40 from skin allergy (including dermal, ocular, etc.) (Table 1).

Dust samples collected from 57% houses of Assam showed mites population >50 per 500 mg dust. Out of them, 17% showed population more than 100/500 mg dust. Among the selected patients, dust sample collected from patient number 118 (204.0/500 mg) followed by patients number 31 (187.0/500 mg), 13 (152.0/500 mg), and 39 (122.0/500 mg) were found to have maximum mites population in their dust samples. Nonetheless, the average dust produced in most of the houses survey range from 4 to 5 g per day. When we categorized the samples on the basis of building types, maximum mite population was recorded in samples collected from RCC types of building (46%) followed by Assam type semi-RCC building (36%). Lowest population was found in the dust sample collected from wooden house. *Acarus, Blomia, Cheyletus,* and *Dermatophagoides* were the dominant genus found in RCC type building, while in Assam type building, *Acarus* and *Dermatophagoides* were recorded maximum. Bomboo house showed *Acarus, Blomia,* and *Cheyletus* while wooden house showed *Acarus* and *Dermatophagoides* as common dust mite (Table 2). Large numbers of hard cell dust mites (which are not considered for the present study) were visible in the samples collected from bamboo and wooden house as compared to other housing pattern.

**Figure 1:**
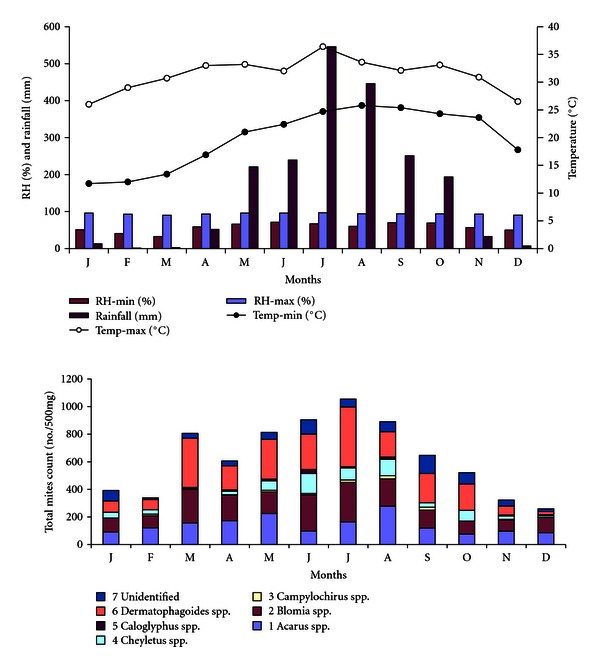
Climatogram of the study sites for the period of January to December 2005 and the monthly distribution of house dust in the indoor dust of selected patients house.

**Figure 2:**
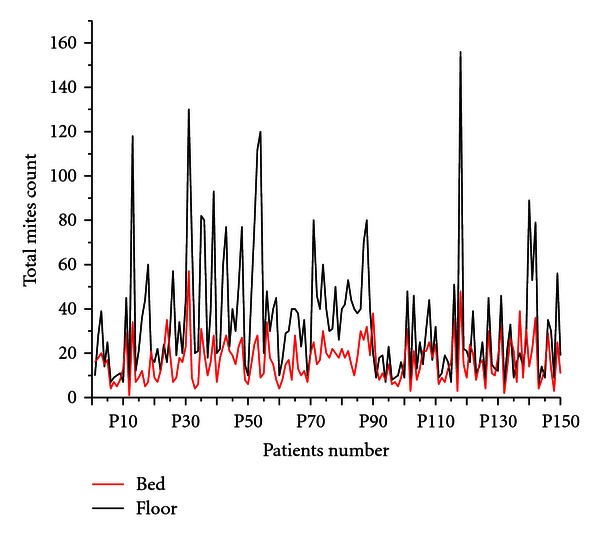
Distribution of dust mites in floor and bed dust samples of selected patients house.

Seasonal studies on the distribution of dust mites showed maximum concentration between June to August during which the atmospheric temperature, relative humidity, and rainfall were maximum, and it accounts for 37.7% of total mite population (Figure 1). *Acarus* spp. was found maximum during the month of August (278/500 mg) followed by May (224/500 mg), whereas, *Blomia* spp. was maximum during the month of July (286/500 mg) followed by June (263/500 mg) and March (243/500 mg), respectively. Similarly, *Dermatophagoides* spp. was found maximum during the month of July (434/500 mg) followed by March (356/500 mg). Least population was recorded from November to February, that is, winter season. Indoor dust mite counts showed significant correlation with the monthly mean rainfall (*r* = 0.783, *P* < .05) and monthly mean maximum temperature (*r* = 0.775, *P* < .05) while insignificant positive correlation with relative humidity. The level of correlation between mites count and temperature, humidity, rainfall is represented by linear regression equation (Figure 2).

Out of the total mites identified, 38.7% were male and 27.9% were female, while 33.3% of the dust mites could not be categorized due to indistinct structure. Maximum male mites were collected from the patients of the age group between 30–50 yrs age group while maximum female was recorded in the house of the age group between 1–30 yrs age group. 

Six different genus of house dust mites were identified in the present survey. The dominant genus was *Dermatophagoides spp*. (2341), followed by *Blomia spp.* (1936), *Acarus spp*. (1686), and *Cheyletus spp*. (671). *Acarus*, *Blomia,* and *Dermatophagoides* were found dominant in several house dust surveyed at different concentration (Table 3). Among *Dermatophagoides, D. farinae *(849) followed by *D. pteronyssinus* (733) was found to be the dominant species. Similarly, *A. siro* and *B. tropicalis* were the identified species of the genus *Acarus* and *Blomia, *respectively. 

**Figure 3:**
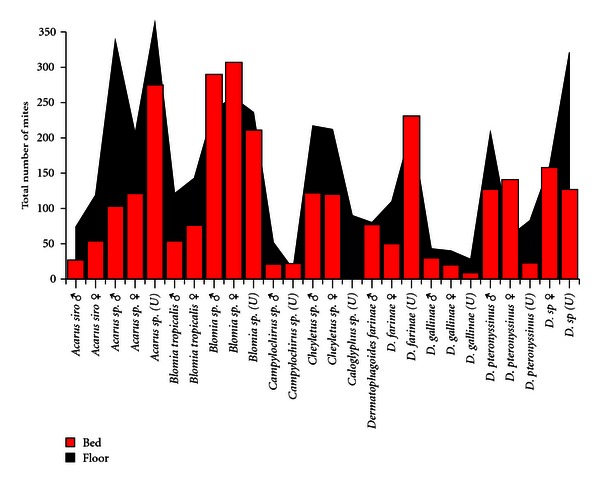
Distribution of different mites species collected and identified from floor and bed dust samples from allergic patients house.

**Figure 4:**
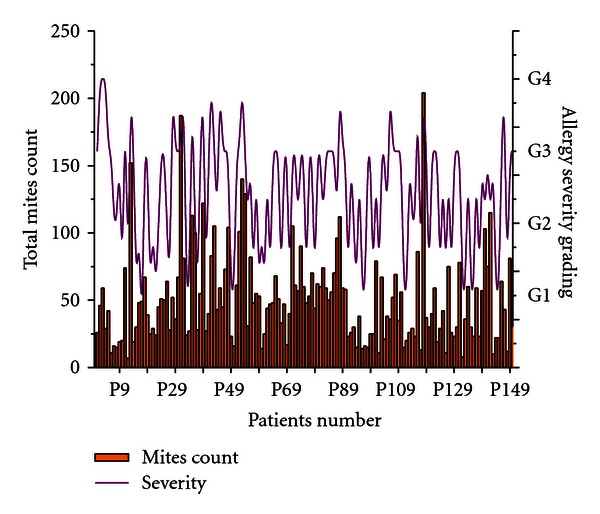
Total number of dust mites collected per 500 mg of dust samples from selected allergic patients in Assam, showing the correlation between mites population and severity of allergic/asthma attack.

About 12% of the patients showed higher mites population in the bed sample as compared to the floor dust and the rest 88% patients showed higher mites concentration in the floor dust (Figure 3). *Blomia tropicalis* and *Dermatophagoides farinae* were found higher in the bed as compared to floor dust. *Dermatophagoides pteronyssinus* female was also found higher in the bed dust sample, while the other species are found higher in the floor dust samples (Figure 4). 

**Figure 5:**
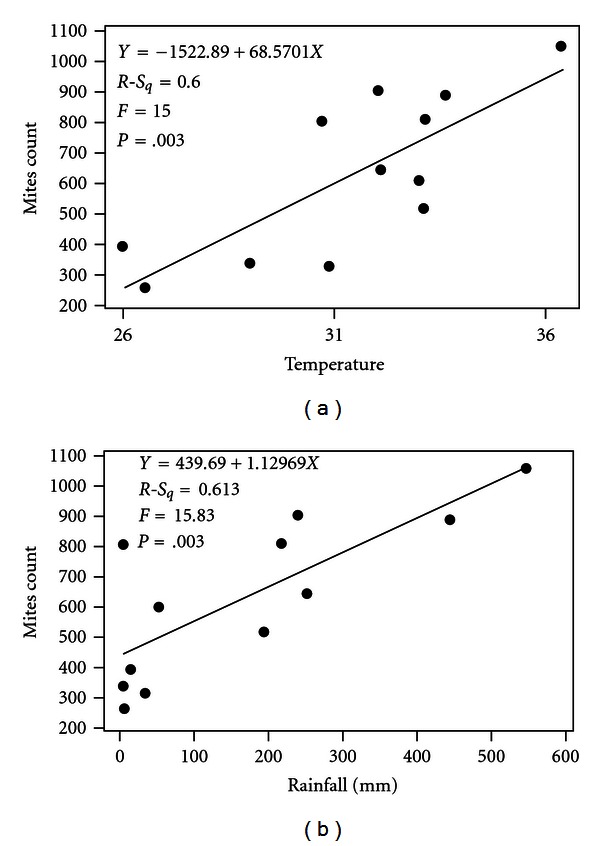


Allergen load of mite in the dust samples collected from the patient's house was found to be proportional to the severity of allergic attack in some of the typical cases. G3 level of severity was recorded in maximum number of cases (73 cases) followed by G1 level (42 cases). G2 and G4 cases were less in number (18 and 17 cases, resp.). About 35% of the patients with G4 level of severity showed higher dust mites count (greater than 100 mites/500 mg of dust sample). Similarly 8% of patients with G3 level of severity were found to have more than 100 mites count/500 mg of dust samples. Maximum number of dust mites were recorded in the samples collected from patient number 118 (204/500 mg dust) followed by P31 (187/500 mg dust), P13 (152/500 mg dust), and all these patients were suffering from frequent respiratory allergic attack (Figure 5). Statistical correlation between the grade of allergic/respiratory symptoms and mites population in the dust samples of the selected samples were found to be significant (*r* = 0.52, *P* < .05). 

## 4. Discussion

Dominance of *Dermatophagoides, Blomia, *and* Cheyletus* in dust samples are well reported from different parts of the worlds [8, 15, 19]. In a survey of homes with dust mites, 60% of the dust mite population is reported from the bed, mattresses, and pillows, 30% in upholstery, and 10% in carpet [20]. Concentration and varieties of mites in the dust samples collected from selected patients house were found to vary from indoor to indoor, which could be due to the difference in the structure and materials of the buildings, socioeconomic status of the individuals, type of mattresses used, standard of hygiene maintained, and difference in the microclimatic conditions that contributes to the higher accumulation of mites in the house dust [21]. Carpet used in RCC and semi-RCC type building may favour higher mites growth [22, 23]. Similarly, jute begs used as mat in bamboo and wooden house in most of the Indian villages might prefer higher dust mites growth. 

The allergenic proteins responsible for causing symptoms are contained not only within the mites themselves but also in their shed skins and especially in their excreta. A single dust mite can produce up to 20 fecal pellets per day, and it may produce approximately 2000 fecal pellets during its active lifetime of up to 3 or 4 months [24]. Adult dust mites can live upto 3 years and can produce excreta about 200 times of their body weight during the life span [25]. Routine human activity such as house cleaning, walking or playing on carpeting, or making the bed, causes the tiny dead/live mites or fecal particles to become airborne and inhaled. Many of these house dust mites such as *Dermatophagoides, Blomia,* and so forth, and their excreta are known to be allergenic to human health [26]. Spieksma [27] and Voorhorst et al. [28] proved potent allergenicity of mites in the house dust and confirmed that extracts of faecal pellets were as potent as those of the mite cultures and these contribute to the allergenicity of house dust. Among the different genus of dust mites recorded, *Dermatophagoides*, particularly *D. farinae* and *D. pteronyssinus, *are known to be highly allergenic to susceptible person [29, 30]. Miyamoto et al. [30] has identified *Dermatophagoides farinae* as a causative agent in bronchial asthma. Present study showed higher *Dermatophagoides farinae* population in the bed dust sample as compared to other dust mites. 

Beds are a prime habitat for dust mites (where 1/3 of life occurs), and a typical used mattress may have upto 10 million mites inside [3]. Mites prefer warm, moist surroundings such as the inside of a mattress when someone is on it. Humans shed about 1/5 ounce of dander (dead skin) each week, which is one of the favorite mite's food (both human and animal skin flakes). About 80 percent of the material seen floating in a sunbeam is actually skin flakes. Also, bedroom carpeting and household upholstery support high mite populations [3]. 

The number of allergenic mites in the bed and floor dust of the patients is well correlated with intensity of the skin test reactions [31, 32]. Allergen load of mites collected above from the dust samples were found directly proportional to the severity of allergic attack in some of the typical cases. Simplício et al. [33] reported *Dermatophagoides farinae* (Der f1) as major allergens in bedding dust samples and stated that Der f1 represents an important risk factor for exacerbation of allergic symptoms in previously mite-sensitized guests. 

As recorded in the present study, higher mites population is encountered during the summer and early autumn months [22]. Mites population are generally recorded maximum during the rainy seasons, but their effect are found maximum during the dry and cooler months of the year [17]. Andrews et al. [34] concluded that mite positive patients had aggravation of respiratory symptoms in the colder months when mites were dead and had disintegrated to dust and had no seasonal changes in symptoms. Patients allergenic to house dust had aggravation to allergic rhinitis and asthma in the fall months when mite number was greater in their cities [35]. Physical interventions (namely, steam cleaning plus vacuuming and vacuuming alone) offer practical, effective means of reducing house dust mite allergen levels in low-income, urban home environments [23]. Change in dwelling accompanied by treatment gives the patient better relief in mite's allergic patients [27]. 

## Figures and Tables

**Table 1:** Classification of patients on the basis of sex and allergic disorders. Total mites population in the house of different age group of patients are also revealed.

Age group	No. of Patients (%)		Allergic symptoms		Dust mites recorded		
	Male	Female	Respectively (%)	Skin (%)	Male	Female	Unidentified sex
1–30 yrs	36	7	27	16	936	810	834
>30–50 yrs	55	22	45	33	1116	751	795
>50 yrs	18	11	18	11	875	547	887

**Table 2:** Distribution of mites in the dust samples collected from different houses of South Assam.

House type	No. of patients (%)	Mites population	Dominant genus
RCC	69	3574	*Acarus, Blomia, Cheyletus, Dermatophagoides*
Assam Type	55	2848	*Acarus, Dermatophagoides*
Bamboo house	17	786	*Acarus, Blomia, Cheyletus*
Wooden house	9	343	*Acarus, Dermatophagoides*

**Table 3:** Total mites population of different genus and their distribution in indoor houses.

S. No.	Mites types	Total number	Houses showing maximum dust mites population
1	*Acarus spp.*	1686	13, 31, 43, 53, 54, 88
2	*Blomia spp.*	1936	13, 35, 39, 54, 71, 88, 118
3	*Campylochirus spp.*	109	39, 54
4	*Cheyletus spp.*	671	31, 39, 48, 118
5	*Caloglyphus spp.*	90	48, 142
6	*Dermatophagoides spp.*	2341	13, 31, 35, 53, 54, 71, 118
7	Unidentified	718	35, 36, 43, 118, 142
